# The different biological effects of TMPyP4 and cisplatin in the inflammatory microenvironment of osteosarcoma are attributed to G‐quadruplex

**DOI:** 10.1111/cpr.13101

**Published:** 2021-07-23

**Authors:** Jianqiang Chen, Xiangxiang Jin, Yanan Mei, Zhe Shen, Jufan Zhu, Hongyi Shi, Minshan Wang, Xiaohui Zheng, Guang Liang

**Affiliations:** ^1^ Chemical Biology Research Center School of Pharmaceutical Sciences Wenzhou Medical University Wenzhou China; ^2^ The Affiliated Xiangshan Hospital Wenzhou Medical University Ningbo China; ^3^ Hospital of Chinese Medicine of Haishu District Ningbo China; ^4^ School of Pharmaceutical Sciences Hangzhou Medical College Hangzhou China

**Keywords:** cisplatin, G‐quadruplex, inflammatory microenvironment, osteosarcoma, TMPyP4

## Abstract

**Objective:**

Osteosarcoma (OS) is characterized by high levels of the tumour‐associated inflammatory microenvironment. Moreover, in approximately 60% of OS, telomere length is maintained by alternative lengthening of telomeres (ALT) pathway. Whether the ALT pathway can be exploited for OS therapeutic treatment and how the OS inflammatory microenvironment influences the anti‐cancer drug effect remains unknown. Here, we examined the biological effects of TMPyP4 and cisplatin in the inflammatory microenvironment of OS cells.

**Materials and methods:**

Immunofluorescence in situ hybridization (IF‐FISH) and C‐circle experiments were used to detect the G‐quadruplex and ALT activity. The redox potential of single guanine, G‐quadruplex and G‐quadruplex/TMPyP4 was evaluated by the lowest unoccupied molecular orbital energy (LUMO), zeta potential and cyclic voltammetry. Cell viability, flow cytometry and apoptosis, Western blot, comet assay, adhesion, transwell and scratch experiments were performed to compare the anti‐tumour proliferation and migration effects of TMPyP4 and cisplatin in the inflammatory microenvironment.

**Results:**

This study indicated that compared with cisplatin, TMPyP4 could induce the formation of human telomeres and FAK G‐quadruplex in vitro and in vivo, and TMPyP4‐treated OS cells showed fewer extrachromosomal C‐circles and fewer ALT‐associated promyelocytic leukaemia bodies. Consequently, the ALT activity and FAK‐related cell migration were suppressed by TMPyP4. Mechanistically, the formation of G‐quadruplex resulted in both lower redox potential than G within the genome and FAK transcription inhibition, and TMPyP4 could enhance this phenomenon, especially in the inflammatory microenvironment.

**Conclusions:**

Our results reveal that TMPyP4 is more suitable for OS treatment than cisplatin.

## Graphical abstract

Osteosarcoma is one of the most malignant cancers with high mortality rates. TMPyP4, a photosensitizer and G‐quadruplex stabilizer, and cisplatin possess anti‐tumour pharmacological effect on various cancer cells. Up to now, however, there is no literature report on the anti‐osteosarcoma effect of TMPyP4 or cisplatin in the inflammatory microenvironment. In our study, we found that TMPyP4 has better anti‐osteosarcoma effect than cisplatin in the inflammatory environment, and their different biological effects are attributed to G‐quadruplex.

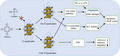

## 1 INTRODUCTION

Currently, osteosarcoma (OS) is a highly aggressive bone tumour that most commonly affects children and adolescents.^1^, ^2^ Surgical resection of tumours followed by chemotherapy constitutes the current standard procedure for clinical OS therapy due to its relative resistance to radiotherapy.^3^ However, OS is often refractory to standardized chemotherapy regimens, and the application of tumour chemotherapy drugs has many adverse effects.^4^, ^5^ In recent decades, several efforts have been made to develop OS therapy, including new pharmacological findings for novel drugs such as sorafenib (Nexavar)^6^ and nanomedicines that aim to release chemotherapeutic drugs in local sites.^7^, ^8^ Despite all medical advances, treatment and outcomes for OS have remained unchanged over the past 30 years, with a 5‐year survival rate below 30%.^9^, ^10^ Thus, uncovering the factors that reduce the therapeutic effects of chemotherapy on OS has important significance.

Local tumours in OS patients are often accompanied by excessive inflammation characterized as ‘red and swollen as well as hot and pain’, and this tumour‐associated inflammatory microenvironment is closely related to the high morbidity, poor outcomes and mortality of OS in the clinic.^11^ This local severe inflammation of OS infiltrated by macrophages is also considered one of the markers of OS.^12^, ^13^ Although the mechanisms are unclear, clinicians and scholars have found that the levels of inflammation are correlated with tumour resistance to treatment.^14^, ^15^ Additionally, chemotherapy further promotes inflammatory events^16^ and induced inflammation seems to play a role in the proliferation, angiogenesis and metastasis of OS.^17^ Consequently, the inflammatory microenvironment of OS together with chemotherapy‐induced inflammation could minimize the clinical efficacy of chemotherapy, even causing its failure and enhancing the invasion and migration of OS cells, leading to death.^17^, ^18^ Therefore, finding a new treatment strategy is vitally important for the treatment of OS.

Additionally, OS is distinct from most cancers in that the majority of OS lack telomerase activity and use the alternative lengthening of telomeres (ALT) mechanism to maintain telomeres.^19^ However, anti‐cancer drugs targeting ALT are still unavailable,^20^ which is another challenge of OS treatment in addition to the severe inflammatory microenvironment and represents a large obstacle in OS treatment. Currently, mechanistic evidence suggests a model in which ALT is mediated by endogenous homologous recombination (HR) machinery.^21^ Our previous study suggested that the formation of G‐quadruplex in the 3′‐terminus of single‐stranded telomeric DNA could prevent the invasion/annealing of telomeric ssDNA and, therefore, have potential value as an anti‐ALT cancer therapeutic.^22^ In this regard, a chemotherapy drug endowed with both potent anti‐tumour effects and specifically targeting telomeric G‐quadruplex might have more advantages in OS treatment.

TMPyP4, which possesses a strong electron‐hole transfer capability, is a novel type of synthetic water‐soluble photosensitizer in photodynamic therapy (PDT).^23^ Compared with normal tissues, TMPyP4 is easily enriched in tumour tissues and, therefore, has a stronger tumour targeting ability.^24^ Recently, TMPyP4 was reported to stabilize G‐quadruplex both in vitro and in vivo.^25^, ^26^, ^27^ Since G has the lowest redox potential within the genome,^28^ the formation of G‐quadruplex might constitute a dominant site for electrons and/or redox,^29^ and TMPyP4 could enhance this phenomenon.^27^ Redox is the essential controlling factor of inflammation because activating adequate immune response cells requires sufficient and rapidly available energy resources, which are intrinsically linked with the redox state.^30^ It is a reasonable assumption that TMPyP4 would be more suitable for OS treatment than most clinical chemotherapy drugs, such as cisplatin.

In this report, we provide evidence that TMPyP4 has more advantages than most clinical chemotherapy drugs in OS treatment, especially in ALT‐positive OS cells, in the inflammatory microenvironment. The results showed that the inflammatory microenvironment can enhance the sensitivity of ALT‐positive OS U2OS and SAOS‐2 cells to TMPyP4 while reducing the anti‐cancer effect of cisplatin and promoting OS cell migration. The implications of these results are discussed.

## 2 MATERIALS AND METHODS

### 2.1 Drug administration

TMPyP4 (Item Number: 323497) and LPS (Item Number: L6386) were purchased from Sigma‐Aldrich with purity ≥97%. Cisplatin was from Selleck (Item number: S1166). TNF‐α was from Peprotech (Item number: 315‐01A). TMPyP4 was dissolved in water, and cisplatin was dissolved in dimethyl sulfoxide (DMSO) for storage and further diluted to final concentrations.

### 2.2 Cell viability assay

Cell viability was assessed with the 3‐(4,5‐dimethylthiazol‐2‐yl)‐2,5‐diphenyltetrazolium bromide (MTT) assay, according to a previously published protocol.^31^ Cells (3000 cells/well in 96‐well plates) were incubated at 37°C for 6 hour and then treated with TMPyP4 (10 μmol L^–1^) or cisplatin (5 μmol L^–1^) for 24 hour followed by the addition of TNF‐α (10 ng/mL) or LPS (1 μg/mL) for 48 hour (total effect 72 hour), then MTT at 0.5 mg/mL per well for another 4 hour. The reaction product formazan was dissolved in 100 μL of DMSO after discarding the culture medium. Cell viability was determined by reading the absorbance at 490 nm with an American thermoelectric Thermo Fisher Multiskan FC automatic microplate reader. The result is expressed as the mean ±standard deviation of three measurements (n = 3).

### 2.3 Immunofluorescence‐FISH assay

Immunofluorescence (IF) was performed as previously described.^32^ Cells on coverslips were fixed with 4% paraformaldehyde for 15 minutes, washed with PBS three times, permeabilized in 0.5% Triton ×‐100 in PBS for 30 minutes and then incubated with blocking solution (5% goat serum in 1 × PBS) for 1.5 hours at room temperature. Cells were loaded with primary antibodies in PBST against BG4 (Sigma), 53BP1 (CST), FAK (CST) or phalloidin (CST) overnight at 4°C. Cells were washed with PBST three times and then incubated with DyLight 488‐conjugated anti‐rabbit or DyLight549‐conjugated anti‐rabbit secondary antibodies for 1.5 hours at room temperature. The coverslip was washed with PBST six times, fixed with 4% paraformaldehyde for 30 minutes, washed with PBS, dehydrated with graded ethanol, incubated with PNA probe, denatured at 85°C for 5 minutes and hybridized overnight at 37°C. The cells were washed and mounted with DAPI and imaged using a Nikon Ti microscope.

### 2.4 C‐circle assay

A C‐circle assay was performed as described previously.^33^ Briefly, U2OS and SAOS‐2 cell samples were harvested, and genomic DNA was extracted according to the instructions in the AxyPrep Blood Genomic DNA Miniprep Kit. The concentration was detected by a NanoDrop. Next, 1 μg of genomic DNA was mixed with Rsa I, HinfI (4 U/μg) and RNase A (25 ng/μg) to configure a 20 μL digestion system and digested at 37°C overnight. Then, 10 μL of digestion product diluted with TE to 50 μL, and 1 μL of the diluted sample were mixed with 10 μL of the C‐Circle reaction system (0.1% Tween, 0.2 mg/mL BSA, 1 mmol L^–1^ dTTP, dGTP, dATP each, 0.5 U Φ29 DNA polymerase and 1×Φ29 buffer) and water, which was amplified at 30°C for 8 hours followed by 65°C for 30 minutes. The product was diluted to 60 μL with 2 × SSC solution, blotted onto an NC membrane and subjected to UV cross‐linking. Subsequently, the membrane was exposed to a phosphor screen and scanned. The results were quantified using Image Q software.

### 2.5 The calculation of LUMO energy

First, PDB (https://www.rcsb.org) was used to establish the initial geometric structure of the single‐stranded nucleic acid sequence (TTAGGG)_4_ and G‐quadruplex. Second, quantum chemistry calculations at a density functional theory of ωB97X‐D/6‐31G(d) were used to fully optimize the simplified models. The lowest unoccupied molecular orbital (LUMO) energies of a single G, G4 and TMPyP4‐G4 were calculated by Gaussian09.

### 2.6 Zeta potential measurements

The hTel oligomer was resuspended in 10 mmol L^–1^ Tris‐HCl (pH 7.4) that contained 100 mmol L^–1^ KCl or no metal cations. The concentration of the oligomer was 3 μM, and TMPyP4 was dissolved at 10 mmol L^–1^ in water for later use. Next, the oligomers were heated at 90°C for 5 minutes, slowly cooled to RT (this process is best to perform overnight in a heat preservation device to prevent too‐rapid cooling) and then incubated at 4°C before the experiment. The zeta potential was determined using a Zetasizer Nano ZS apparatus (Malvern Instruments). All measurements were tested in triplicate (n = 3).

### 2.7 Electrochemical measurements

Cyclic voltammetry (CV) measurements were performed with a CHI 1030b electrochemical workstation (Shanghai Chenhua Co.). Conventional three‐electrode tests were performed at RT with a glassy carbon electrode (GCE) used as the working electrode, a saturated calomel electrode (reference electrode) and a platinum electrode serving as the auxiliary electrode. Among them, the working electrode was polished with metallographic sandpaper before each measurement, polished with an Al_2_O_3_ suspension and then washed with ethanol and pure water ultrasonically for use. The electrochemical test electrolyte solution was 10 mmol L^–1^ Tris‐HCl (pH 7.4) or 10 mmol L^–1^ Tris‐HCl (pH 7.4) that contained 100 mmol L^–1^ KCl buffer solution, and the potential scanning range was from −2.5 V to 1 V at a rate of 5 mV S^‐1^. It is worth noting that nitrogen was passed through to remove oxygen for more than 1 hour before the experiment, and the experimental process was carried out under nitrogen.

### 2.8 Circular dichroism measurements

Circular Dichroism (CD) spectra were recorded using a spectropolarimeter (Applied Photophysics Ltd., UK) with a 1 cm long quartz cell, the wavelength range of 200‒390 nm, and 200 nm min^−1^ scan speed with three acquisitions at room temperature. The oligomer of FAK was resuspended in 10 mmol L^–1^ Tris‐HCl (pH 7.4) that contained 100 mmol L^–1^ KCl or no metal cations. The concentration of the oligomer was 10 μmol L^–1^, and TMPyP4 was dissolved in 10 mmol L^–1^ water for later use. Subsequently, the oligomers were heated at 90°C for 5 minutes, slowly cooled to RT (this process is best to stay overnight in a heat preservation device to prevent too fast cooling) and then incubated at 4°C before the experiment. The buffer baseline correction is measured in the same quartz cell. During the titration experiment, the oligomer was fixed to 10 μmol L^–1^, and different concentrations of TMPyP4 were added and equilibrated for at least 10 minutes (until there is no change in the CD signal) before performing spectral scanning. Data analysis was carried out by using GraphPad Prism 5.0.

### 2.9 Quantitative real‐time RT‐PCR analysis

TRIzol (Invitrogen) was used to extract total RNA from cultured OS cells. cDNA was produced using a reverse transcription reagent kit (TaKaRa) according to the manufacturer's instructions. Reverse transcription and quantitative PCR were performed using Power SYBR Green qPCR master mix (Invitrogen) in a LightCycler 480 (Roche) with the following specific primers (Thermo Fisher): GAPDH, 5′‐GCACCGTCAAGGCTGAGAAC‐3′ (forward) and 5′‐TGGTGAA GACGCCAGTGGA‐3′ (reverse); FAK, 5′‐AGTAAAATCCAGCCAGCCCC‐3′ (forward) and 5′‐GACATACTGCTGGGCCAGTT‐3′ (reverse). The housekeeping gene glyceraldyde‐3‐phosphate dehydrogenase (GAPDH) was used for normalization.

### 2.10 Western blotting

Cells were washed, lysed and boiled for 10 minutes. Proteins were separated by 12% SDS‐PAGE gels, transferred to a 0.25 μm PVDF membrane, blocked in 5% dry milk in TBST for 1.5 hours and incubated with primary antibodies against FAK (CST), p‐FAK (CST), γ‐H2AX (Millipore), RPA (CST) and β‐actin (CST) overnight at 4°C. The membranes were washed, incubated with the secondary antibody for 1.5 hour and detected using a Westar Supernova kit (Cyanagen) with a Gel Imager System (Bio‐Rad).

### 2.11 Cell adhesion assay

The cell adhesion assay was performed as described previously.^34^ At room temperature, we coated a 96‐well plate with 2.5 μg/mL human fibronectin in PBS (Millipore). Then, the treated cells were seeded into serum‐free medium at a density of 4 × 10^4^ cells/well and cultured at 37°C for 30 minutes under 5% CO2. Cells treated with vehicle (0.1% DMSO) were used as controls. The medium was gently removed, and then, the cells were fixed with 4% paraformaldehyde and stained with crystal violet at room temperature for 5 minutes. After dissolving the crystal violet with 100 μL DMSO, the absorbance was measured at 560 nm. The following formula was used to calculate the relative number of cells attached to the extracellular matrix: average of treated cells OD/average OD control unit. The relative number of cells attached to the extracellular matrix was calculated using the following equation: (mean OD of treated cells/mean OD of control cells) ×100%.

### 2.12 Transwell assay

Transwell assays were performed as described previously.^34^ Treated U2OS and SAOS‐2 cells were digested, resuspended and diluted with serum‐free media to a concentration of 5 × 10^5^/mL. Transwell chambers were added to a 24‐well plate; subsequently, 600 μL of 10% FBS was added to the lower chamber. After 100 μL of prepared cell suspension was pipetted from each group into the upper chamber, and the 24‐well plate was cultured in a 37°C incubator. After 24 hour, fluid and cells in the chamber were discarded, and the cells were washed 3 times with pre‐warmed PBS. After fixing with 4% paraformaldehyde for 15 minutes at room temperature, the cells on the chamber membrane were washed 3 times with PBS and then stained with crystal violet for 5 minutes in the dark. The product was dissolved in 33% glacial acetic acid and then measured by reading the absorbance at 490 nm with the American thermoelectric Thermo Fisher Multiskan FC automatic microplate reader. Cell migration rate: average of treated cells OD/average OD control unit) ×100%.

### 2.13 Scratch‐wound assay

Cell migration was detected by the scratch‐wound assay. In short, U2OS and SAOS‐2 cells were treated with or without drug, seeded in 6‐well plates and grown to confluence in the growth medium. The wound was scratched by a sterile 200 μL pipette tip approximately 1 mm wide on the cell layer. Subsequently, pictures of the wound were captured by inverted microscopy at the same position at different times (0, 24, 48, 72 and 96 hour), and the scratched area was measured by ImageJ. All experiments were performed at least three times.

### 2.14 Cell cycle distribution analysis

U2OS and SAOS‐2 cells were pre‐treated with TMPyP4 (10 μmol L^–1^) or cisplatin (5 μmol L^–1^) for 24 hour, followed by the addition of TNF‐α (10 ng/mL) or LPS (1 μg/mL) for 48 hours (total effect 72 hours), collection, trypsinization, washing and fixation with 1 mL 70% cold ethanol at 4°C overnight. The cells were centrifuged, washed with PBS and then loaded with 0.05 mg/mL propidium iodide (PI) for 10 minutes at room temperature. The cell cycle distribution was tested with flow cytometry (BD FACSCalibur, BD Biosciences).

### 2.15 Annexin V/PI apoptosis assay

U2OS and SAOS‐2 cells were seeded in 6 cm2 dishes at a density of 2.0 × 10^5^ cells per dish and incubated at 37°C for 6 hour until cells attached to the dish‐treated drug for the indicated time. Subsequently, cells were harvested for the Annexin V/PI apoptosis assay. The assay was performed following the protocol provided by the Annexin V/PI apoptosis kit (Sigma) and assessed with a flow cytometer (BD FACSCalibur, BD Biosciences).

### 2.16 Comet assay

A comet assay was used to detect DNA damage.^35^ Briefly, U2OS cells were mixed with 0.5% low‐melting‐temperature agarose before being transferred onto slides, which were coated with 1.5% normal agarose. For the alkaline assay, the slides were lysed in 1% Triton ×‐100, 10 mmol L^–1^ Tris (pH 10.0), 100 mmol L^–1^ EDTA, 2.5 M NaCl overnight at 4°C and then electrophoresed in 1 mmol L^–1^ EDTA and 300 mM NaOH at 2 V/cm for 15 minutes. The slides were washed with water, dried with ethanol, mounted with PI solution (20 μg/mL) and captured under a fluorescence microscope (Nikon Ti microscope).

### 2.17 Statistical analysis

The student's two‐tailed unpaired *t* test was used to determine the statistical significance and the resulting *P*‐values are indicated in figures (*p < .05; **p < .01; ***p < .001).

## 3 RESULTS

### 3.1 TMPyP4 induces the formation of G‐quadruplex and inhibits the ALT activity of OS cells

**FIGURE 1:**
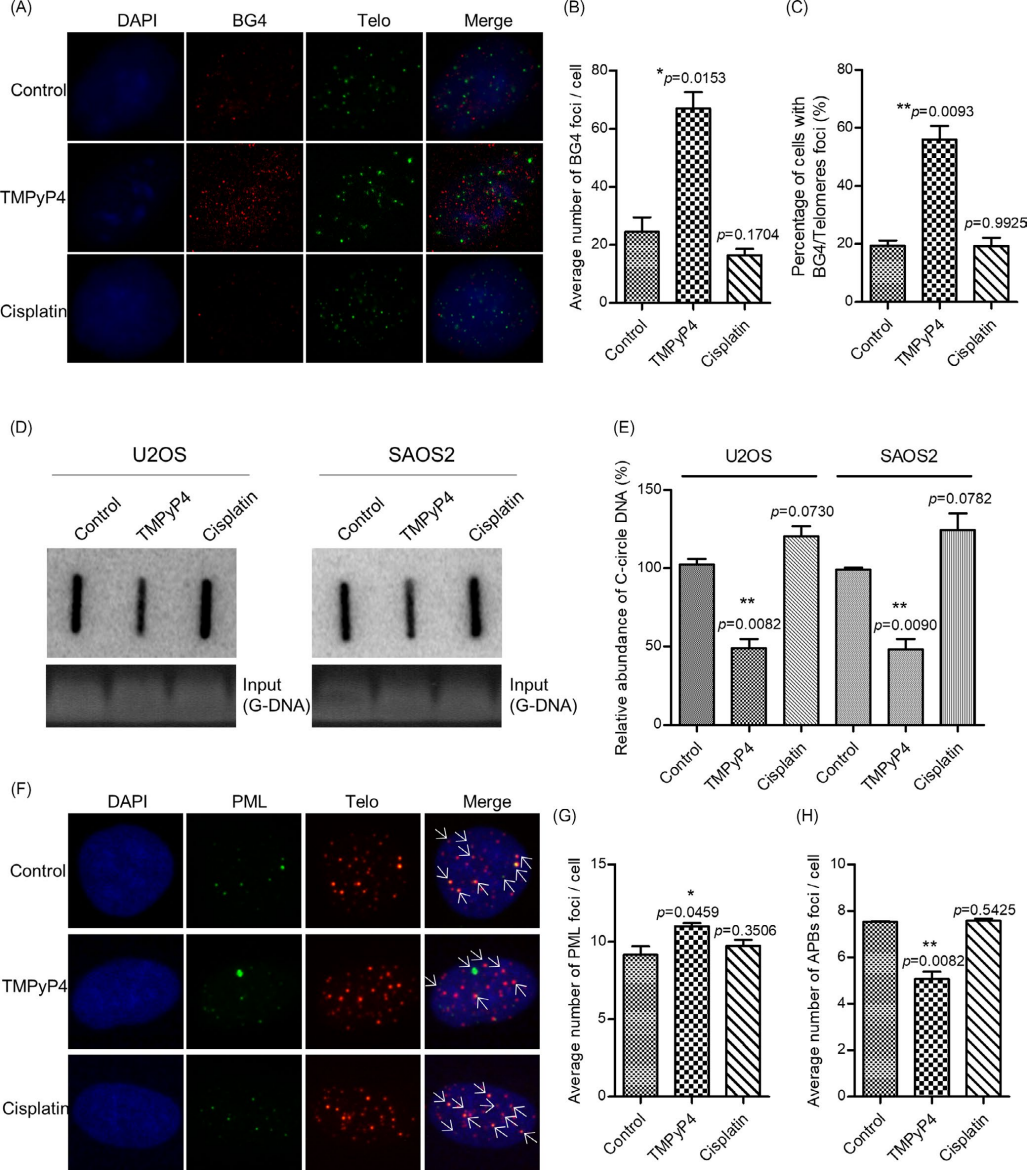
TMPyP4 induces the formation of G‐quadruplex in vivo and inhibits the ALT activity of osteosarcoma U2OS and SAOS‐2 cells. (A) Immunofluorescence (IF) and fluorescence in situ hybridization (FISH) were used to detect G‐quadruplex or telomeric G‐quadruplex in U2OS cells treated with 10 μmol L^–1^ TMPyP4 or 5 μmol L^–1^ cisplatin for 48 h. Antibodies against G‐quadruplex and cy3‐labelled telomeric probes were used to visualize the telomeres. Magnification: 400×. (B) and (C) Quantification of A. (D) C‐circle assay in drug‐treated U2OS and SAOS‐2 cells. Cells were treated with 10 μmol L^–1^ TMPyP4 or 5 μmol L^–1^ cisplatin for 48 h. Ethidium bromide staining (bottom) served as a control for equal loading. E, Quantification of D. (F) IF and FISH was used to detect the formation of ALT‐associated promyelocytic leukaemia (PML) bodies in U2OS cells. Cells were treated with TMPyP4 and cisplatin for 72 h. Antibodies against PML and cy3‐labelled telomeric probes were used to visualize PML bodies and telomeres, respectively. Arrows indicate colocalized foci. Magnification: 400×. (G) and (H) Quantification of F. For each group, 200 or more cells were examined, and values are the average ± SD of three independent experiments. The statistical significance was calculated using the unpaired Student's two‐tailed *t* test (*p < .05, **p < .01, ***p < .001)

It is well known that TMPyP4 stabilizes G‐quadruplex in vitro^26^ and may promote the formation of G‐quadruplex in vivo. To test this hypothesis, immunofluorescence using antibodies to G‐quadruplex (BG4)^36^ was performed to visualize G‐quadruplex in U2OS and SAOS‐2 cells. In ALT‐positive U2OS and SAOS‐2 cells, 48 hour of treatment with TMPyP4 (10 μmol L^–1^) significantly increased the number of BG4 foci, which on behalf of G‐quadruplex, while cisplatin at 5 μmol L^–1^ slightly decreased the number of G‐quadruplex (Figure 1A, Figure S1A). The obtained results demonstrated that TMPyP4 promotes the formation of G‐quadruplex and telomeric G‐quadruplexes, while the cross‐linking between cisplatin and G nucleobases suppresses this process (Figure 1B‐1C, Figure S1B‐1C).

The formation of G‐quadruplex could result in a variety of cellular consequences, including altered gene expression, impaired DNA replication and/or cell cycle arrest.^26^ Additionally, our previous report demonstrated that the formation of telomeric G‐quadruplex inhibits ALT activity.^22^ ALT was characterized by the presence of extrachromosomal C‐circle DNA and the formation of ALT‐associated PML bodies (APBs) and a high frequency of HR at telomeres.^20^ Using a previously characterized assay for C‐circle DNA,^22^ we observed less C‐circle DNA in TMPyP4‐treated U2OS cells and SAOS‐2 cells (Figure 1D‐1E). The number of APBs also decreased in TMPyP4‐treated U2OS and SAOS‐2 cells but did not change after cisplatin treatment (Figure 1F‐1H, Figure S1D‐1F). These results indicated that TMPyP4 treatment suppresses ALT activity by inducing the formation of telomeric G‐quadruplex.

### 3.2 The G‐quadruplex has a lower redox potential than a single guanine base, and TMPyP4 can enhance this phenomenon

**FIGURE 2:**
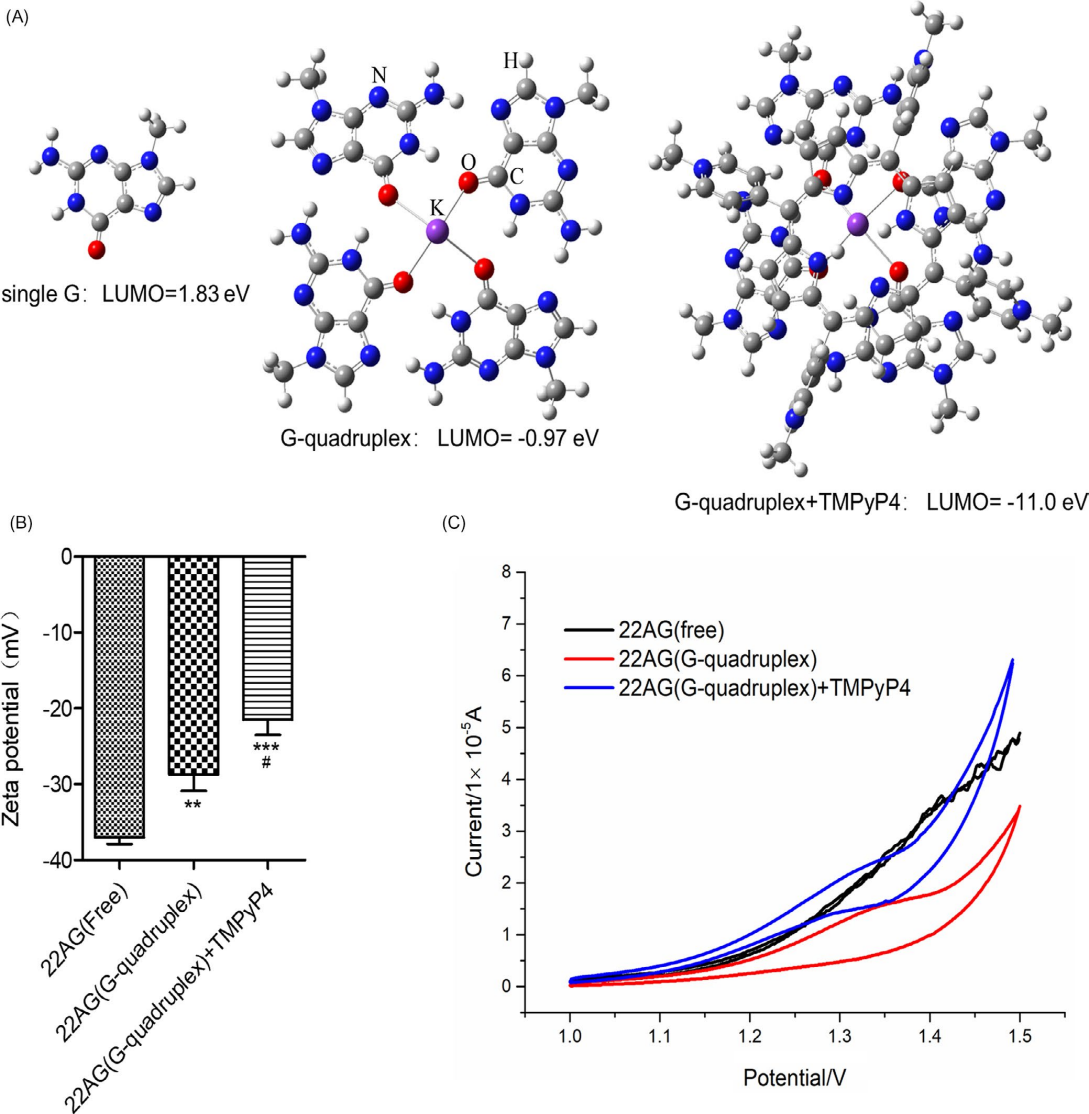
The G‐quadruplex has a lower redox potential than a single guanine base, and TMPyP4 can enhance this phenomenon. (A) The lowest unoccupied molecular orbital (LUMO) energy of a single G, G‐quadruplex and G‐quadruplex/TMPyP4. The simplified models were fully optimized by quantum chemistry calculations at the density functional theory of ωB97X‐D/6‐31G(d). The calculations were completed in Gaussian09. (B) The zeta potentials of G‐oligos, G‐quadruplex and G‐quadruplex/TMPyP4. Indicated values are mean ± SD (n = 3). #, *p* < .05, compared with 22AG (G‐quadruplex). (C) Cyclic voltammograms of G‐oligo, G‐quadruplex and G‐quadruplex/TMPyP4. The statistical significance was calculated using the unpaired Student's two‐tailed *t* test (*p < .05, **p < .01, ***p < .001)

As mentioned before, a single G has the lowest redox potential within the genome, and the formation of a G‐quadruplex might be a dominant site for electrons and/or redox; therefore, we first calculated the lowest unoccupied molecular orbital (LUMO) energy of a single G, G‐quadruplex and G‐quadruplex/TMPyP4. Quantum chemistry calculations at the density functional theory of ωB97X‐D/6‐31G(d)^37^ were used to fully optimize the simplified models. The LUMO energies of a single G, G‐quadruplex and G‐quadruplex/TMPyP4 were calculated to be 1.83, −0.97 and −11.0 Ev (Figure 2A), respectively. The results showed that the LUMO energy of single G was lower after the formation of the G‐quadruplex. After binding with TMPyP4, the LUMO of G‐quadruplex/TMPyP4 further decreased to −11.0 eV, suggesting that the TMPyP4‐stabilized G‐quadruplex was more conducive to accepting electrons and exhibited a stronger oxidation ability and a more positive reduction potential through Gaussian09.^38^

It is expected that the surface properties of the G‐oligo might be changed by the formation of a G‐quadruplex structure and might be further affected by TMPyP4. The zeta potential of the G‐oligo was −37.03 ± 0.63 mV, while the zeta potentials of the G‐quadruplex and G‐quadruplex/TMPyP4 decreased to −28.7 ± 1.33 mV and −20.46 ± 1.53 mV (Figure 2B), respectively. The results suggested that the formation of G‐quadruplex reduced the negative charge density of the nucleic system and that TMPyP4 further reduced the negative charge density, making G‐quadruplex and/or G‐quadruplexes/TMPyP4 more vulnerable to electrons than G‐oligos.

Moreover, cyclic voltammetry (CV) assays further determined that compared with G‐oligo, G‐quadruplex and/or G‐quadruplex/TMPyP4 was confirmed to pass the current more effectively and observed a more obvious oxidation peak (Figure 2C). This is consistent with the findings of quantum chemistry calculations and zeta potential tests.

### 3.3 TMPyP4 was endowed with stronger anti‐cancer activity in the inflammatory microenvironment

**FIGURE 3:**
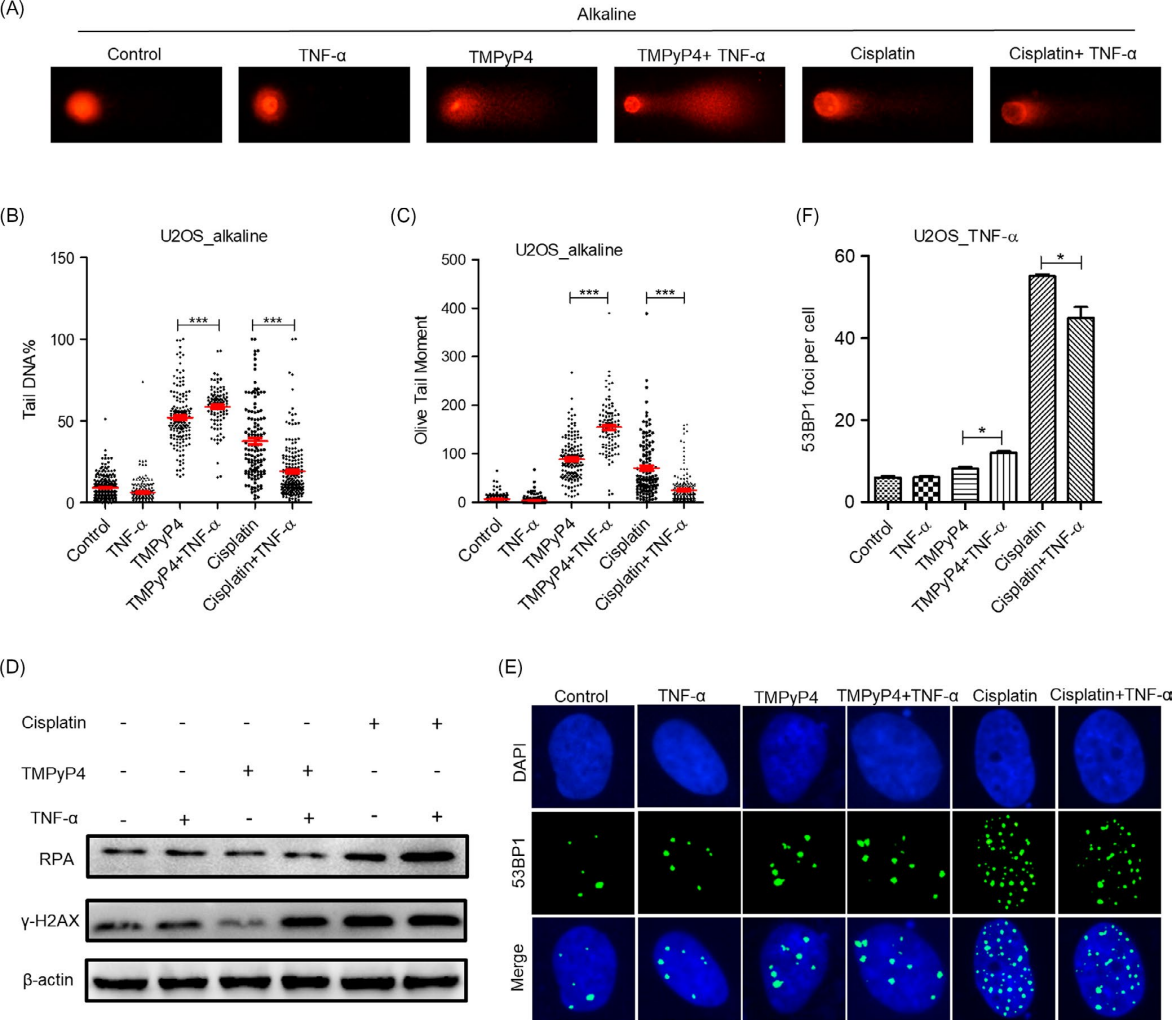
TMPyP4 triggers intense DNA damage and provokes a strong DNA damage response in the inflammatory microenvironment. (A) Representative results of alkaline comet assay. U2OS cells were pre‐treated with 10 μmol L^–1^ TMPyP4 or 5 μmol L^–1^ cisplatin for 24 h, 10 ng/mL TNF‐α was added to the wells for 48 h and ≥200 cells were examined in each group. Magnification: 400×. (B) and (C) Quantification of A. (D) Western blot analysis of γ‐H2AX and RPA in U2OS cells exposed to 10 μmol L^–1^ TMPyP4 or 5 μmol L^–1^ cisplatin for 24 h and then treated with 10 ng/mL TNF‐α to the appropriate wells for 48 h. β‐actin was used as a control. (E) Immunofluorescence assay was used to determine the 53BPl foci in drug‐treated U2OS cells at the indicated time. Magnification: 400×. (F) Quantification of data in E, ≥200 cells were examined in each group. The values are represented as the mean ± SD of at least three independent experiments. The statistical significance was calculated using the unpaired Student's two‐tailed *t* test (*p < .05, **p < .01, ***p < .001)

The formation of G‐quadruplex triggers intense DNA damage and provokes a strong DNA damage response.^26^ Considering the high level of inflammation in OS ^2^, ^3^ and the importance of inflammation in promoting tumour progression as well as the close correlation between inflammation and DNA damage,^11^, ^12^ we utilized tumour necrosis factor α (TNF‐α) and bacterial lipopolysaccharides (LPS) to induce an inflammatory response to simulate the inflammatory microenvironment of OS in the following experiments. The comet assay indicated that TMPyP4 triggered more DNA damage in OS cells in the inflammatory microenvironment, where cisplatin lost half of the ability to induce DNA damage (Figure 3A‐3D, Figure S2A‐2D). In addition, considering that phosphorylated histone H2AX, γ‐H2AX, is one of the important markers of DNA double‐strand breaks,^39^ and we used WB experiments to detect the expression level of γ‐H2AX. The results showed that compared with conventional culture environment, TMPyP4 induces more γ‐H2AX expression in the inflammatory microenvironment, while cisplatin slightly reduces its expression (Figure 3D, Figure S2D). Meanwhile, the expression of RPA, which coats ssDNA, is a key physiological signal activating DNA damage repair,^40^ but it was not affected by TMPyP4 in the inflammatory microenvironment, while cisplatin increased its expression (Figure 3D, Figure S2D). As expected, TMPyP4 provoked more 53BP1 foci per cell in the inflammatory microenvironment than in the conventional culture environment (Figure 3E‐3F, Figure S2E‐2F). However, cisplatin provoked little DNA damage response under inflammatory microenvironment than conventional culture environment with the number of 53BP1 foci per cell decreased from ~55 to ~42 (Figure 3E‐3F, Figure S2E‐2F).

**FIGURE 4:**
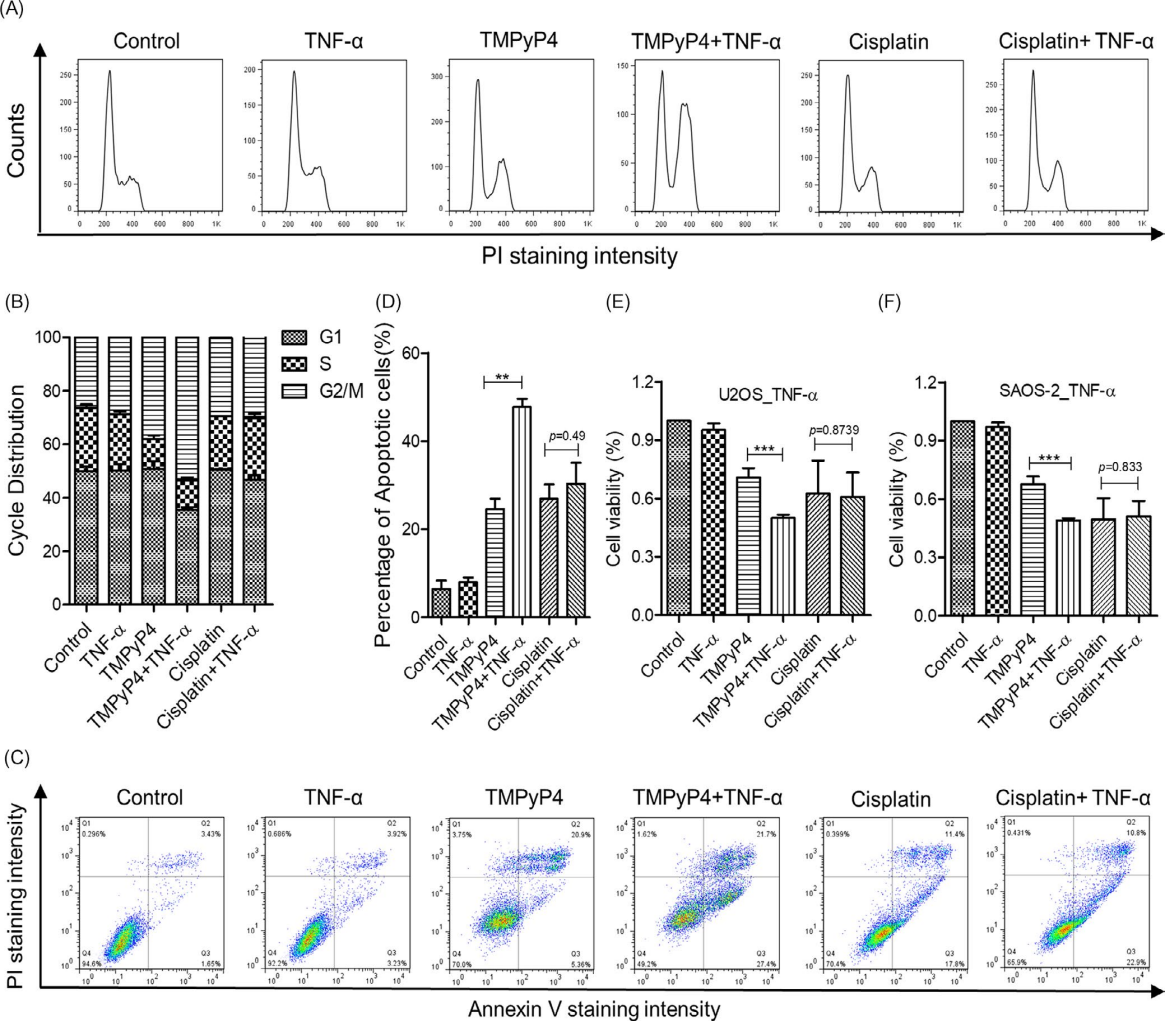
TMPyP4 was endowed with stronger anticancer activity in an inflammatory microenvironment. (A) TMPyP4 blocked more of the cell cycle to G2/M phase in the inflammatory microenvironment. (B) Quantification of cell cycle distribution. (C) TMPyP4 caused the most U2OS cell apoptosis in the inflammatory microenvironment. Cells were exposed to 10 μmol L^–1^ TMPyP4 or 5 μmol L^–1^ cisplatin for 24 h, incubated with 10 ng/mL TNF‐α in the appropriate wells for another 48 h and then collected and assessed by Annexin V/PI staining. (D) Quantification of C. (E) and (F)d, Effect of TMPyP4 or cisplatin on the viability of U2OS and SAOS‐2 cells in the presence or absence of 10 ng/mL TNF‐α for 48 h. The cell viability was measured by MTT. The values are represented as the mean ± SD of at least three independent experiments. The statistical significance was calculated using the unpaired Student's two‐tailed *t* test (*p < .05, **p < .01, ***p < .001)

Consequently, TMPyP4 blocked more of the cell cycle transition to G2/M phase (53%) in the inflammatory microenvironment, whereas the distribution of cell cycle arrest was not affected by cisplatin treatment (Figure 4A‐4B, Figure S3A‐3B). Furthermore, TMPyP4 caused the most cell apoptosis and the lowest cell viability in U2OS and SAOS‐2 cells with TNF‐α or LPS stimulation, compared with the other groups (Figure 4C‐4F, Figure S3C‐3F).

### 3.4 TMPyP4 reduces the pseudopodia area of OS, while cisplatin increases it in the inflammatory microenvironment

**FIGURE 5:**
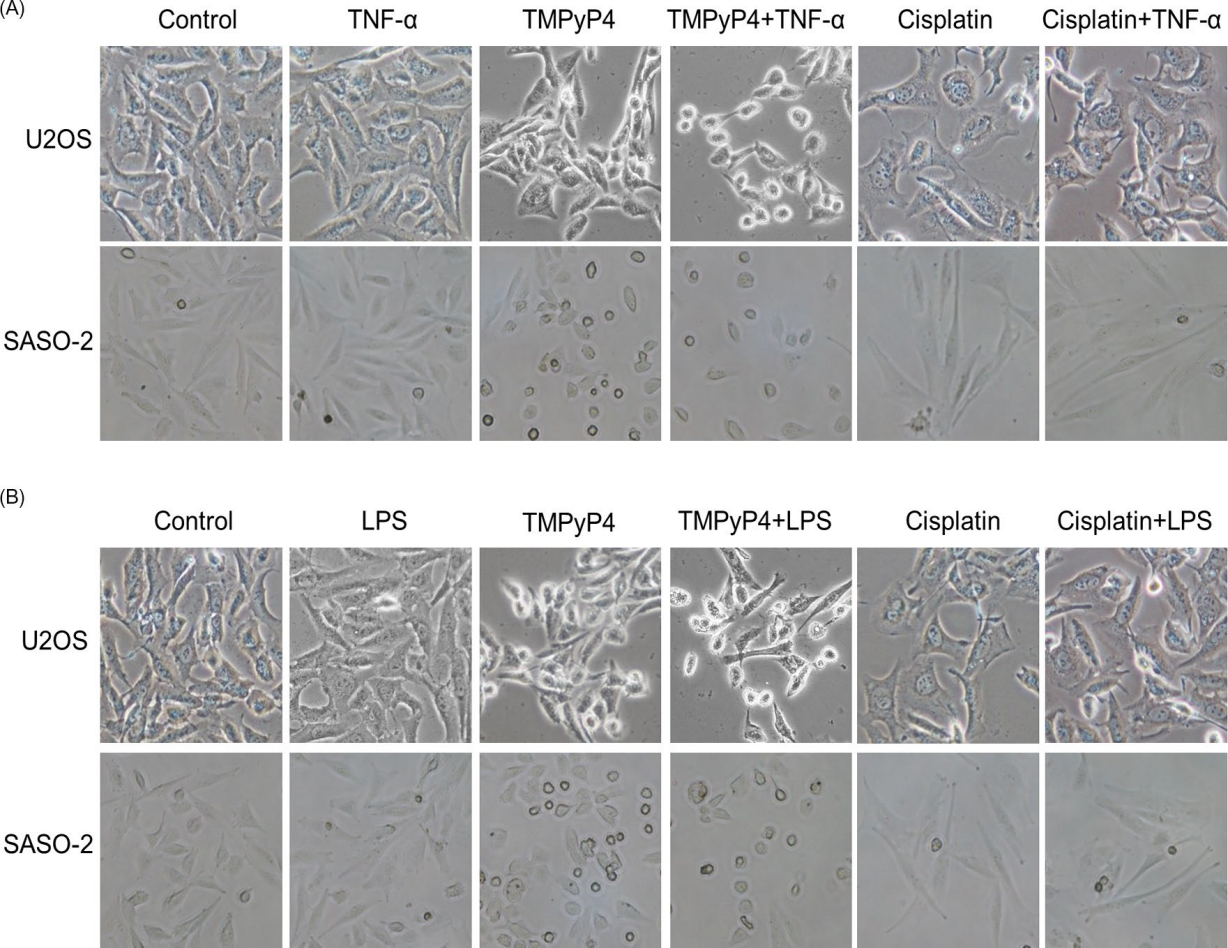
The U2OS and SAOS‐2 cells shrank, decreased round and smooth when challenged with TMPyP4 or cisplatin in the inflammatory microenvironment, while the cisplatin or cisplatin combined with TNF‐α or LPS group became elongated. (A) and (B) Cells were treated with TMPyP4 or cisplatin in the presence or absence of TNF‐α (10 ng/mL) or LPS (1 μg/mL) for the indicated times, and then, phase contrast images were captured. Magnification: 100×

**FIGURE 6:**
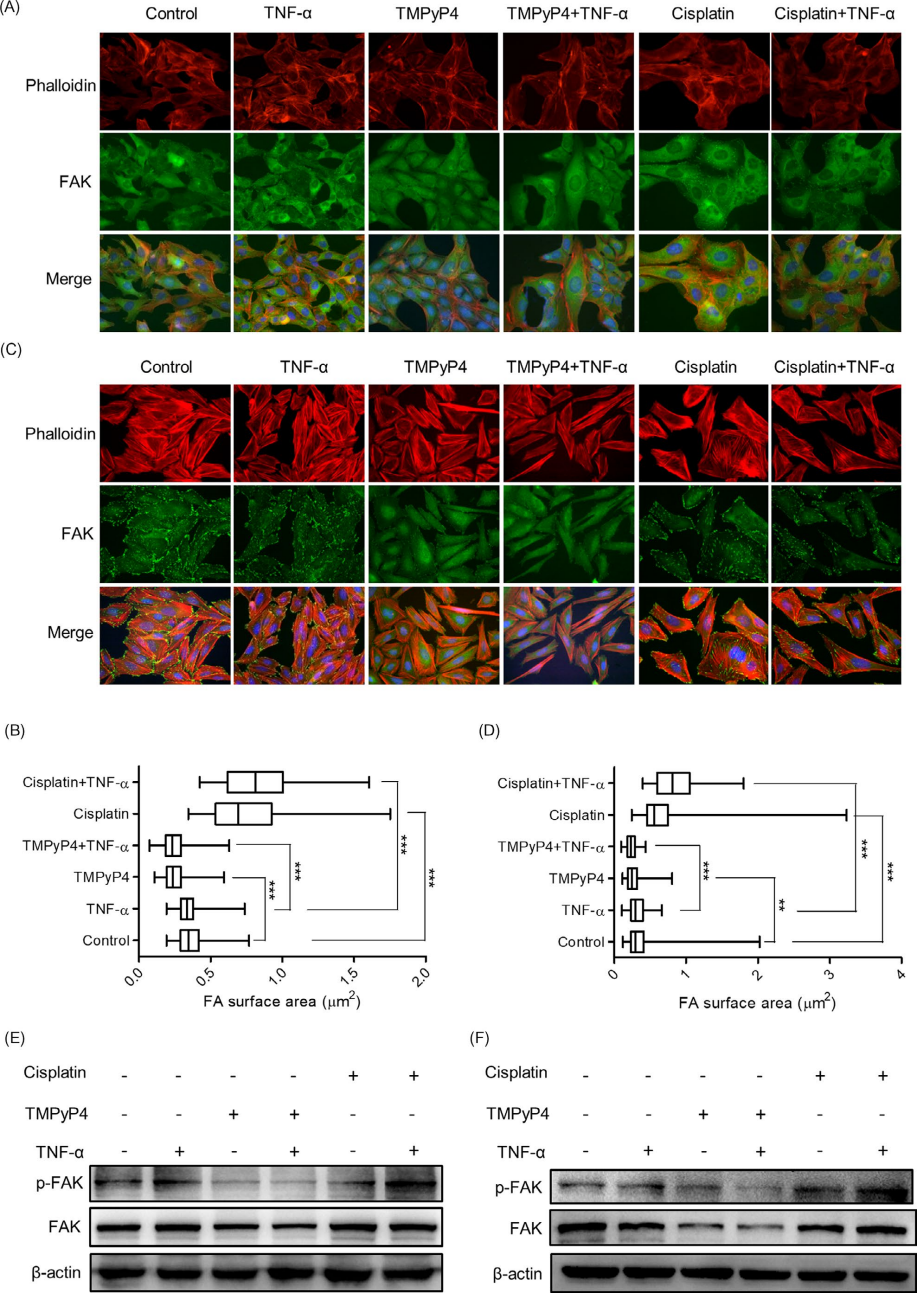
TMPyP4 inhibits the expression and distribution of FAK in OS cells in the inflammatory microenvironment. (A) and (C) Representative images of FAK in U2OS and SAOS‐2 cells pre‐treated with TMPyP4 (10 μmol L^–1^) or cisplatin (5 μmol L^–1^) for 24 h and treated with or without TNF‐α (10 ng/mL) for 48 h in IF assays. Antibodies against FAK (green) and phalloidin (red) were used to visualize FAK and F‐actin, respectively. Magnification: 200×. (B) and (D) Quantification of data in A and C. The focal adhesion surface area was assessed through FAK and phalloidin staining in U2OS and SAOS‐2 cells. (E) and (F) Western blotting of FAK and p‐FAK in OS cells exposed to TMPyP4 (10 μmol L^–1^) or cisplatin (5 μmol L^–1^) with or without the indicated TNF‐α for 48 h. β‐actin used as loading control. Values represent the mean ± SD of at least three independent experiments, and ≥500 cells were counted in each group. The statistical significance was calculated using the unpaired Student's two‐tailed *t* test (*p < .05, **p < .01, ***p < .001)

During the experiments, we observed that compared with the TMPyP4‐treated alone group, cells in the TNF‐α or LPS and TMPyP4 combination group became shrunken and decreased round and smooth (Figure 5). Conversely, cisplatin‐treated cells cultured in a TNF‐α‐ or LPS‐induced inflammatory microenvironment had a more elongated structure, and cell‐cell junctions were more obvious than those cultured in a normal culture environment (Figure 5). The formation of G‐quadruplex would affect the related gene expression.^26^ We speculated that TMPyP4‐induced G‐quadruplex might be located in related cytoskeletal protein regulatory regions and then inhibit the transcription and expression of these proteins. Focal adhesion kinase (FAK) is a cytoplasmic kinase that is essential for cell morphogenesis and migration.^41^ F‐actin is the most abundant protein and a crucial protein for cell stability, morphogenesis and motility.^42^ To explore whether the different cell morphological changes in TMPyP4‐ or cisplatin‐treated OS cells in a TNF‐α‐ or LPS‐induced inflammatory microenvironment were associated with the expression of FAK and F‐actin, an immunofluorescence assay was performed using a FAK antibody and phalloidin antibody, which is a fluorescent dye of F‐actin filaggrin. The results showed that FAK clusters were associated with actin filaments around the cell edge in untreated U2OS and SAOS‐2 cells, and TNF‐α pre‐conditioning had a negligible influence on actin filaments (Figure 6A and 6C). Compared with untreated cells, TMPyP4‐treated cells presented decreased cell spreading areas, reduced pseudopod shrinkage and downregulated expression of FAK and F‐actin protein in both TNF‐α‐present and TNF‐α‐absent cells (Figure 6B‐6F). However, TNF‐α pre‐conditioning had the opposite effect on cisplatin‐treated OS cells (Figure 6B‐6F). Alone with the FAK expression profile, similar changes were observed in phosphorylated FAK (p‐FAK) level (Figure 6E‐6F). This phenomenon was also observed in LPS pre‐conditioning‐treated U2OS and SAOS‐2 cells (Figure S4).

### 3.5 TMPyP4 induced the formation of G‐quadruplex in the FAK promoter and inhibited the transcription of FAK

**FIGURE 7:**
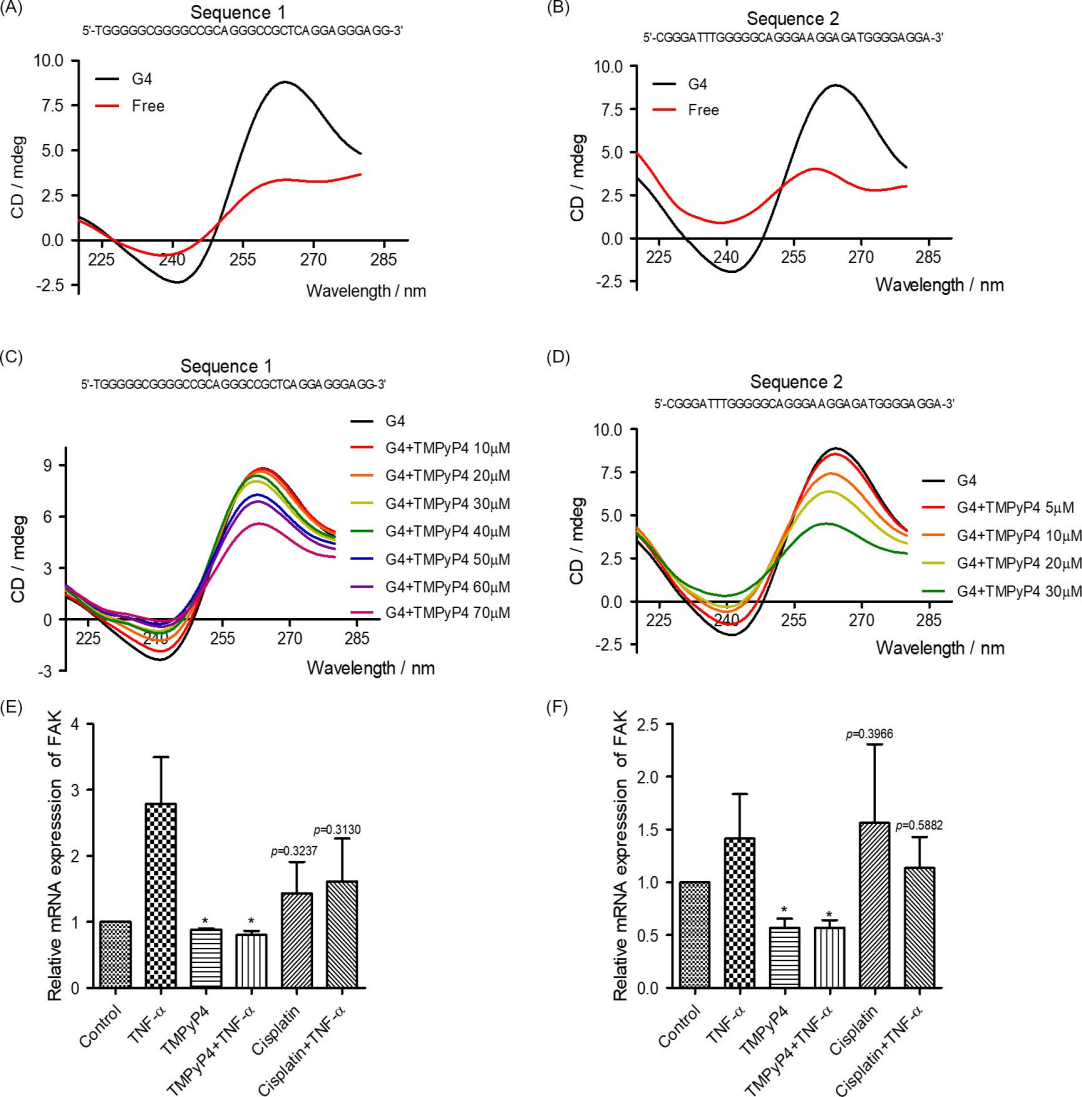
TMPyP4 stabilizes the formation of G‐quadruplex in the FAK promoter. (A) and (B) Selected oligo sequences in the FAK promoter formed a parallel G‐quadruplex, as determined by circular dichroism (CD) spectrometry assays. (C) and (D) CD spectrometry determination of G‐quadruplex in the FAK promoter of selected oligo sequences stabilized by TMPyP4 under near‐physiological conditions (crowding and 100 mmol L^–1^ K+). The conformation of the G4 (FAK promoter G‐quadruplex) sequence (10 μmol L^–1^) was detected by CD spectrometry after incubation with the indicated concentration of TMPyP4 for the indicated times. Sequence 1: TGGGGGCGGGGCCGCAGGGCCGCTCAGGAGGGAGG; Sequence 2: CGGGATTTGGGGGCAGGGAAGGAGATGGGGAGGA. (E) and (F) The mRNA levels of the promoter of FAK in U2OS and SAOS‐2 cells. The cells were pre‐treated with 10 μmol L^–1^ TMPyP4 or 5 μmol L^–1^ cisplatin for 24 h, incubated with 10 ng/mL TNF‐α for 48 h, and then collected for RT‐PCR. The Values are represented as the mean ± SD of at least three independent experiments. The statistical significance was calculated using the unpaired Student's two‐tailed *t* test (*p < .05, **p < .01, ***p < .001)

To explore whether TMPyP4 inhibited the expression of FAK by inducing the formation of G‐quadruplex in FAK‐transcribed genes, we adopted the ‘https://genome.ucsc.edu/’ database to obtain the promoter of PTK2 encoding FAK. Then, the website ‘https://bioinformatics.ramapo.edu/QGRS/index.php’ was used to preliminarily predict the G‐quadruplex formation ability of the PTK2 promoter region. Surprisingly, no less than 11 oligo sequences had G‐quadruplex formation potency (Table S1). Then, a circular dichroism (CD) assay was performed to determine the FAK G‐quadruplex induced by TMPyP4. As expected, we found that the selected FAK oligo sequences would form a parallel G‐quadruplex (a negative peak at 241 nm and a positive peak at 264 nm on CD spectrometry, Figure 7A and 7B), and this structure would stabilize by TMPyP4 under near‐physiological conditions (Figure 7C and 7D). A qPCR assay was further carried out to evaluate the inhibition of FAK transcription by TMPyP4‐induced PTK2 G‐quadruplex. The results showed that the mRNA levels of PTK2 were downregulated in TMPyP4‐ or TMPyP4 plus TNF‐α‐treated OS cells compared with the control or TNF‐α group, while upregulation was observed in the cisplatin or cisplatin combined with TNF‐α group. Taken together, these results indicate that TMPyP4 reduces the pseudopodia area of OS by inducing the formation of persistent G‐quadruplex in PTK2, resulting in the inhibition of FAK expression.

### 3.6 The inflammatory microenvironment endows TMPyP4 with stronger resistance to cell adhesion and migration of OS cells but impairs this ability of cisplatin

**FIGURE 8:**
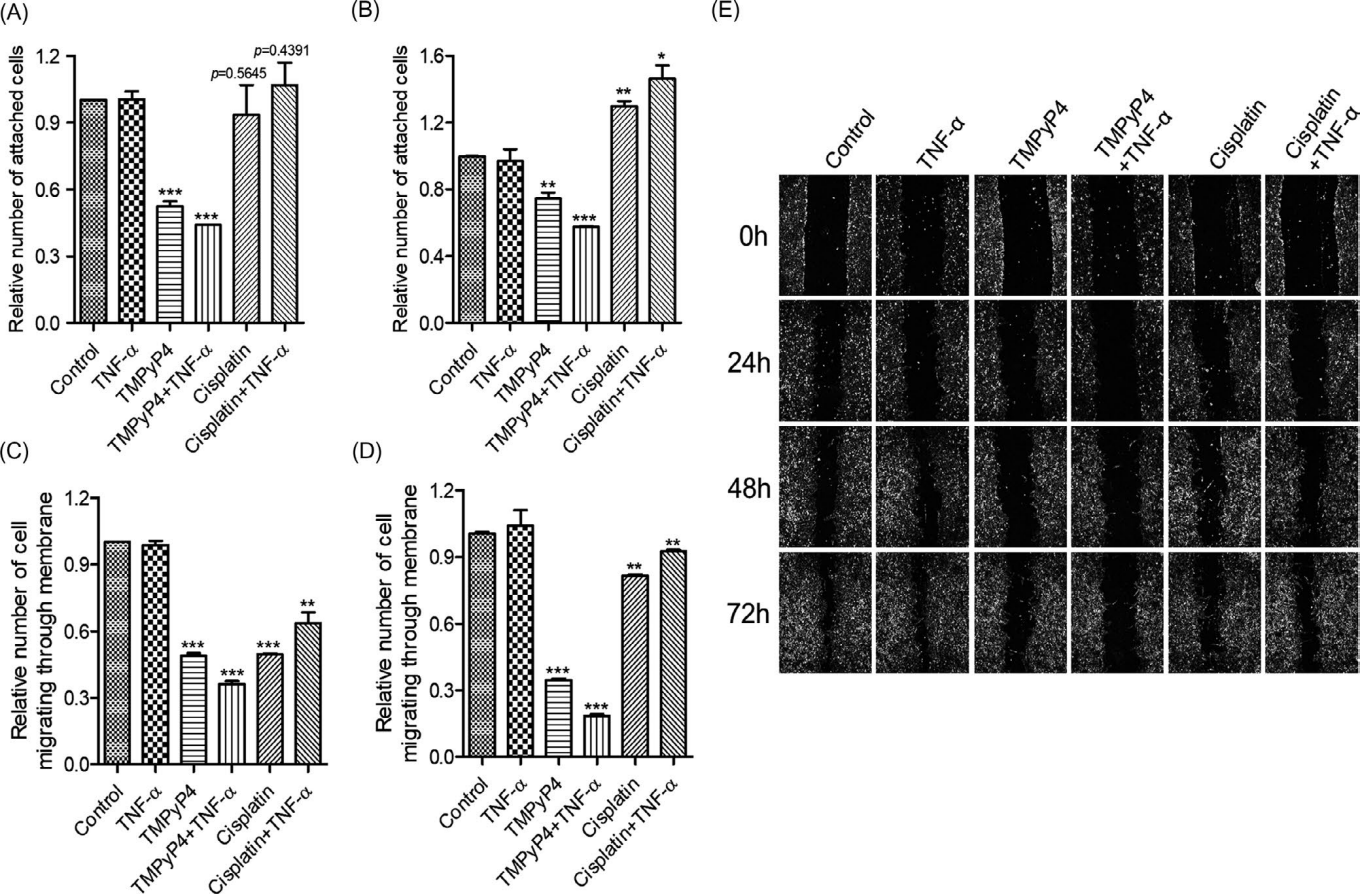
TMPyP4 suppresses the adhesion, invasion and migration of osteosarcoma (OS) cells with or without an inflammatory microenvironment. The cells were pre‐treated with 10 μmol L^–1^ TMPyP4 or 5 μmol L^–1^ cisplatin for 24 h and then incubated with 10 ng/mL TNF‐α for 48 h. (A) and (B) TMPyP4 decreased cell adhesion to the extracellular matrix in U2OS and SAOS‐2 cells in the presence or absence of TNF‐α, while cisplatin increased the adhesion ability of OS cells in the presence of TNF‐α. (C) and (D) TMPyP4 inhibited OS cell migration with or without TNF‐α, as determined by a transwell assay. (E) TMPyP4 inhibits U2OS and SASO‐2 cell migration in scratch‐wound healing assays. Magnification: 40×. The values are represented as the mean ± SD of at least three independent experiments. The statistical significance was calculated using the unpaired Student's two‐tailed *t* test (*p < .05, **p < .01, ***p < .001)

FAK has long been known as a critical regulator of cell migration.^41^ Therefore, a cell adhesion assay together with a transwell assay was first performed to evaluate the effect of TMPyP4 and cisplatin on OS cells and the attached matrix. As shown in Figure 8A‐8D and Figure S5A‐5D, TMPyP4 exhibited stronger cell adhesion to matrix inhibition ability and decreased transferability under TNF‐α‐ or LPS‐induced inflammatory microenvironment compared with conventional culture environment, while this inflammatory environment increased cell adhesion to the matrix and transferability in cisplatin‐treated OS cells. These data suggested that compared with cisplatin, TMPyP4 might have higher potential value in the clinical migration treatment of OS cells.

Cell adhesion is often associated with cell migration.^43^ Next, we performed a scratch‐wound healing assay to determine the migration rate of TMPyP4‐ or cisplatin‐treated U2OS and SAOS‐2 cells in the presence or absence of TNF‐α or LPS. Our results showed that the TNF‐α‐ or LPS‐induced inflammatory microenvironment sensitizes the anti‐migration ability of TMPyP4 but, to some extent, restores the migration ability of cisplatin‐treated cells (Figure 8E, Figure S5E).

## 4 DISCUSSION

Locally higher inflammatory responses, which would limit the effect of chemotherapy,^13^, ^14^ are typical clinical symptoms of OS. Chemotherapy tends to aggravate local inflammatory symptoms, finally resulting in chemotherapy failure.^44^ Furthermore, nearly 40% of OS cell lines are ALT‐positive,^19^ which are resistant to most chemotherapy, and anti‐cancer therapeutics targeting ALT are not yet available, adding extra challenges to OS treatment in the clinic.^9^ The aim of this study was to explore why chemotherapy is stagnant in OS treatment and how to further improve the curative effect. Our results reveal that TMPyP4, a G‐quadruplex stabilizer and photosensitizer, has more advantages in OS treatment than cisplatin in an inflammatory microenvironment.

Cisplatin is a common chemotherapeutic drug that exerts its anti‐cancer effect by producing cross‐linking with guanines (G) in DNA.^45^ Due to the lowest redox potential of G throughout the genome, it has become the preferred target of ROS,^46^ which are produced abundantly during inflammation.^30^ We speculated that cisplatin might not cross‐link with G effectively because of the competitive effect of ROS in the inflammatory microenvironment of OS; thus, the anti‐cancer activity was impaired. Our results revealed that the TNF‐α‐ or LPS‐simulated inflammatory microenvironment increased DNA resistance to cisplatin (Figure 3, Figure S2) and reduced the sensitivity of OS cells to cisplatin, thereby reducing tumour apoptosis and promoting survival (Figures 3 and 4, Figure S3). Our discovery also revealed that cisplatin was ineffective against the migration of OS cells, especially in the TNF‐α‐ or LPS‐simulated inflammatory microenvironment (Figure 8, Figure S5). Mechanistically, cisplatin treatment increased the expression and distribution of FAK, which is attributed to cell migration, at the leading edge of OS cells and promoted the formation of pseudopodia (Figure 6, Figure S4), leading to an increased ability to adhere to the extracellular matrix (Figure 8A and 8B). Accordingly, increased transferability (Figure 8C and 8D) and migration rate (Figure 8E) were observed for cisplatin‐treated cells. It has been widely accepted that increased cell migration and transferability are closely associated with cancer invasion and metastasis, a major cause of osteosarcoma patient death.^47^ Thus, our study implies that cisplatin may increase the risk of OS metastasis.

It has been proposed that G‐quadruplex formation in telomeres inhibits ALT activity and alters gene transcription and expression in the genome. Nearly 40% of OS cells are ALT‐positive,^19^ and G‐quadruplex more easily attract electrons than a single G.^29^ In this regard, a G‐quadruplex inducer and/or stabilizer might have more advantages in OS treatment. TMPyP4 is a photosensitizer in PDT,^27^ and an excellent G‐quadruplex stabilizer^27^ satisfies the above criteria. TMPyP4 induced the formation of telomeric G‐quadruplexes, thereby attenuating C‐circles/APBs, indicating the suppression of ALT (Figure 1, Figure S1). Persistent G‐quadruplex activated stronger DNA damage, which might be exacerbated by ROS generated from inflammation. Consequently, compared with the conventional culture environment, TMPyP4 triggered more intense DNA damage but failed to activate comparable DNA damage responses in the inflammatory microenvironment (Figure 3, Figure S2), leading to cell cycle arrest and apoptosis or senescence (Figure 4, Figure S3).

Migration accounts for therapy failure and cancer‐related death in OS.^2^, ^8^, ^9^, ^43^ In these scenarios, anti‐migration is worthy of more attention in OS treatment. Because G‐quadruplex are widely located at the promoter regions of migration‐related genes,^26^ such as VEGF^48^ and WNT1,^49^ TMPyP4 was expected to inhibit the migration and invasion of OS cells. Consistent with this scenario, compared with cisplatin‐treated OS cells, TMPyP4‐treated OS cells had significantly lower cellular migration and adhesion abilities after only 3 days of treatment, and this inhibitory effect was further strengthened by the TNF‐α‐ or LPS‐induced inflammatory microenvironment (Figure 8, Figure S5). In this study, TMPyP4 induced the formation of persistent G‐quadruplex in PTK2, resulting in the inhibition of FAK expression (Figure 7). Consequently, TMPyP4 effectively suppressed OS cell migration, invasion and cell motility in a TNF‐α‐ or LPS‐induced inflammatory microenvironment (Figures 5 and 8, Figure S5). These results suggested that TMPyP4 has the potential to be an effective anti‐cancer agent of OS and is able to rapidly inhibit the proliferation and migration of OS cells in vivo. Our findings provide a new understanding of the role of G‐quadruplex and TMPyP4 in the treatment of OS in the inflammatory microenvironment.

This study was limited to the inflammatory microenvironment established in this study, which was mainly induced by TNF‐α or LPS, and it could not completely simulate the local OS‐associated microenvironment. Thus, the applicability of TMPyP4 from this study to mouse models, PDX models and patients may be questioned. In addition, TMPyP4‐induced G‐quadruplex are widely present in the genome and could result in a variety of cellular consequences, which might influence the function of normal cells. Our future studies will not only focus on the effect of TMPyP4 on solid tumours, including mouse models and PDX models but also explore stabilizers that specifically induce the formation of G‐quadruplex in metastasis‐related genes and telomeres, which may reduce the risk of adverse off‐target effects in normal human cells.

## CONFLICT OF INTEREST

The authors declare no competing interests.

## AUTHOR CONTRIBUTIONS

GL, XHZ and JQC designed the research. JQC, XXJ, YNM, ZS, JFZ, HYS and MSW performed the research study and analysed the data. XHZ, GL and JQC wrote the paper. All the authors approved the final manuscript and agreed for the publication.

## Supporting information

Supplementary Material

## Data Availability

The data that support the findings of this study are available from the corresponding author upon reasonable request.
