# Supplementary material for: The different biological effects of TMPyP4 and cisplatin in the inflammatory microenvironment of osteosarcoma are attributed to G‐quadruplex
Source: Cell Prolif. 2021 Jul 23;54(9):e13101. doi: 10.1111/cpr.13101 (PMC8450119; doi:10.1111/cpr.13101)
Supplement: Supplementary file 1 — Supplementary Material [file CPR-54-e13101-s001.docx]

**The different biological effects of TMPyP4 and cisplatin in the inflammatory microenvironment of osteosarcoma are attributed to G-quadruplex**

Jianqiang Chen^1^, Xiangxiang Jin^1^, Yanan Mei^1^, Zhe Shen^1^, Jufan Zhu^1^, Hongyi Shi^1^, Minshan Wang^2, 3^, Xiaohui Zheng^1*^, Guang Liang^1,4*^

^1^Chemical Biology Research Center, School of Pharmaceutical Sciences, Wenzhou Medical University, Wenzhou 325035, Zhejiang, China

^2^The Affiliated Xiangshan Hospital, Wenzhou Medical University, Ningbo 315000, Zhejiang, China

^3^Hospital of Chinese Medicine of Haishu District, Ningbo 315000, Zhejiang, China

^4^School of Pharmaceutical Sciences, Hangzhou Medical College, Hangzhou 311399, Zhejiang, China

Correspondence: Xiaohui Zheng ([zhengxh@wmu.edu.cn](mailto:zhengxh@wmu.edu.cn)) or Guang Liang ([wzmcliangguang@163.com](mailto:wzmcliangguang@163.com))

**Supporting Table**

**Supporting Table 1. PTK2 gene promoter can form sequences of G4 structure.**

| Sequence | |
| --- | --- |
| **1** | TGGGGGCGGGGCCGCAGGGCCGCTCAGGAGGGAGG |
| **2** | CGGGATTTGGGGGCAGGGAAGGAGATGGGGAGGA |
| **3** | GTGCGGACTGTGTGTGAGGATAGCAGGGCAGTGGGTGG |
| **4** | CGCGCAAAGGCCTGAGGCTGGCATGGAGAGG |
| **5** | GCGGAAGCCCGGGTCGGTGTCGGGGCGAG |
| **6** | GGTCTGTAGCCCTCGGGAGGGATTGCAGGG |
| **7** | GTTAGGGTCGGTGTGGTGCCTGGAGAGTG |
| **8** | GCAAGGCCCGGGGCCCGCAGCGGGTGCCCGACAGCGGGCG |
| **9** | GCTGGCGGAGAGCGAGGCAGGTGGG |
| **10** | GGATGTCCGCTCGGCGGGGCGACCCCGG |
| **11** | GCGGCGCGCTGGGCATGCGCGGGGGCGGCGCG |

***** The website <https://bioinformatics.ramapo.edu/QGRS/index.php> was used to make a preliminary prediction of the G-quadruplex formation ability of PTK2 promoter region and the 11 oligo sequences would have the G-quadruplex formation potency.

**Supporting Figures**


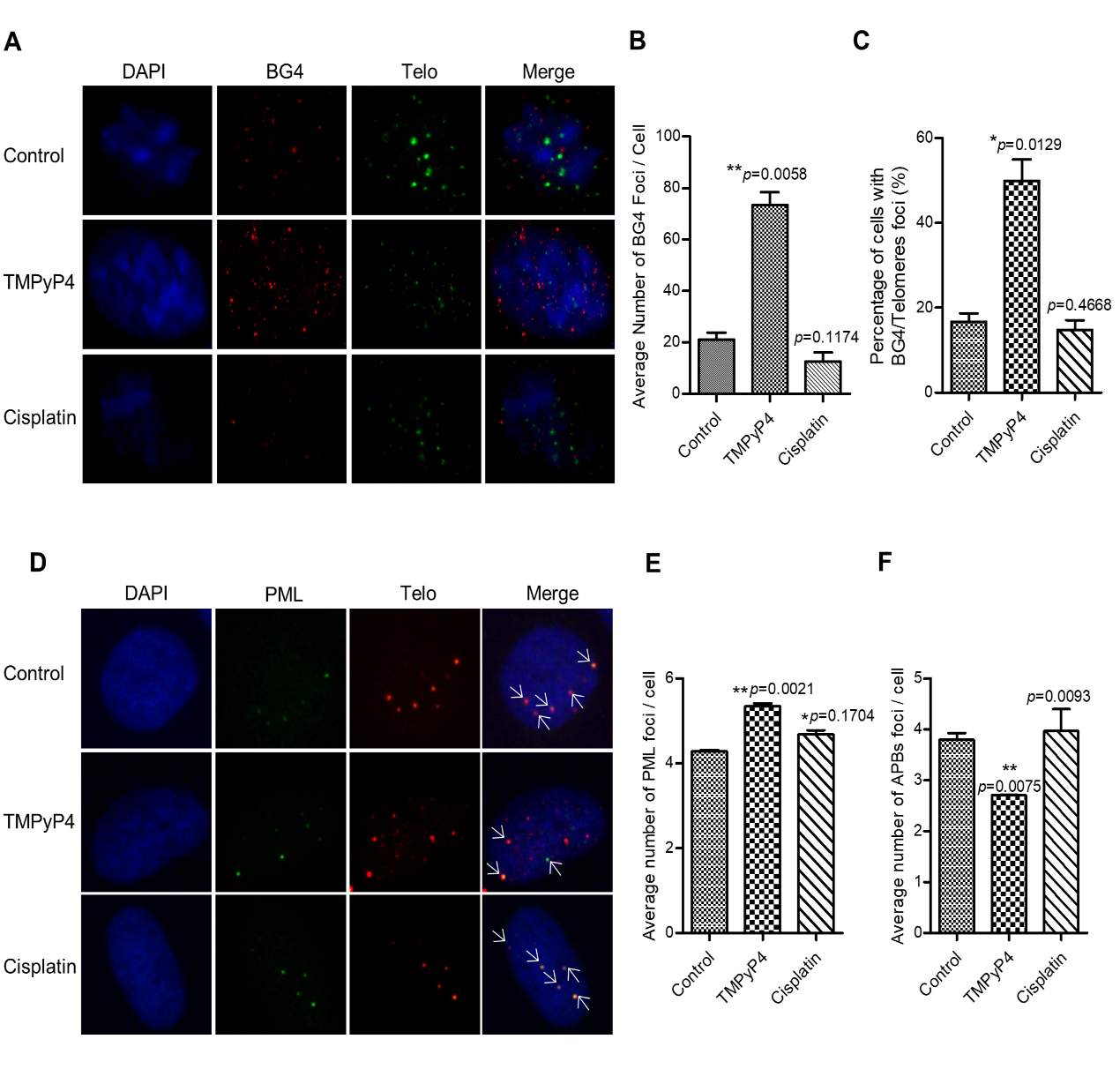


**Supporting Figure 1**. TMPyP4 induces the formation of G-quadruplex and inhibit ALT activity in SAOS-2 cells. Cells were challenged with indicated concentration of TMPyP4 or cisplatin for indicated time. **(A)**, IF&FISH visualization of G4 and telomeres. Magnification: 400×. (B) and (C), Quantification of data in (A). (D) TMPyP4 decreases the amount of APBs in SAOS-2 cells. IF and FISH was used to visualize PML and telomeres, respectively. Magnification: 400×. **(E)** and **(F)**, Quantification of data in (D). For each group, ≥200 cells were examined. Arrows indicate co-localized foci. Values are average ± SD of three independent experiments. p values were calculated using the two-tailed unpaired student’s t-test. The statistical significance was calculated using the unpaired Student’s two-tailed *t*-test (**p* < 0.05, ***p* < 0.01, ****p* <0.001).


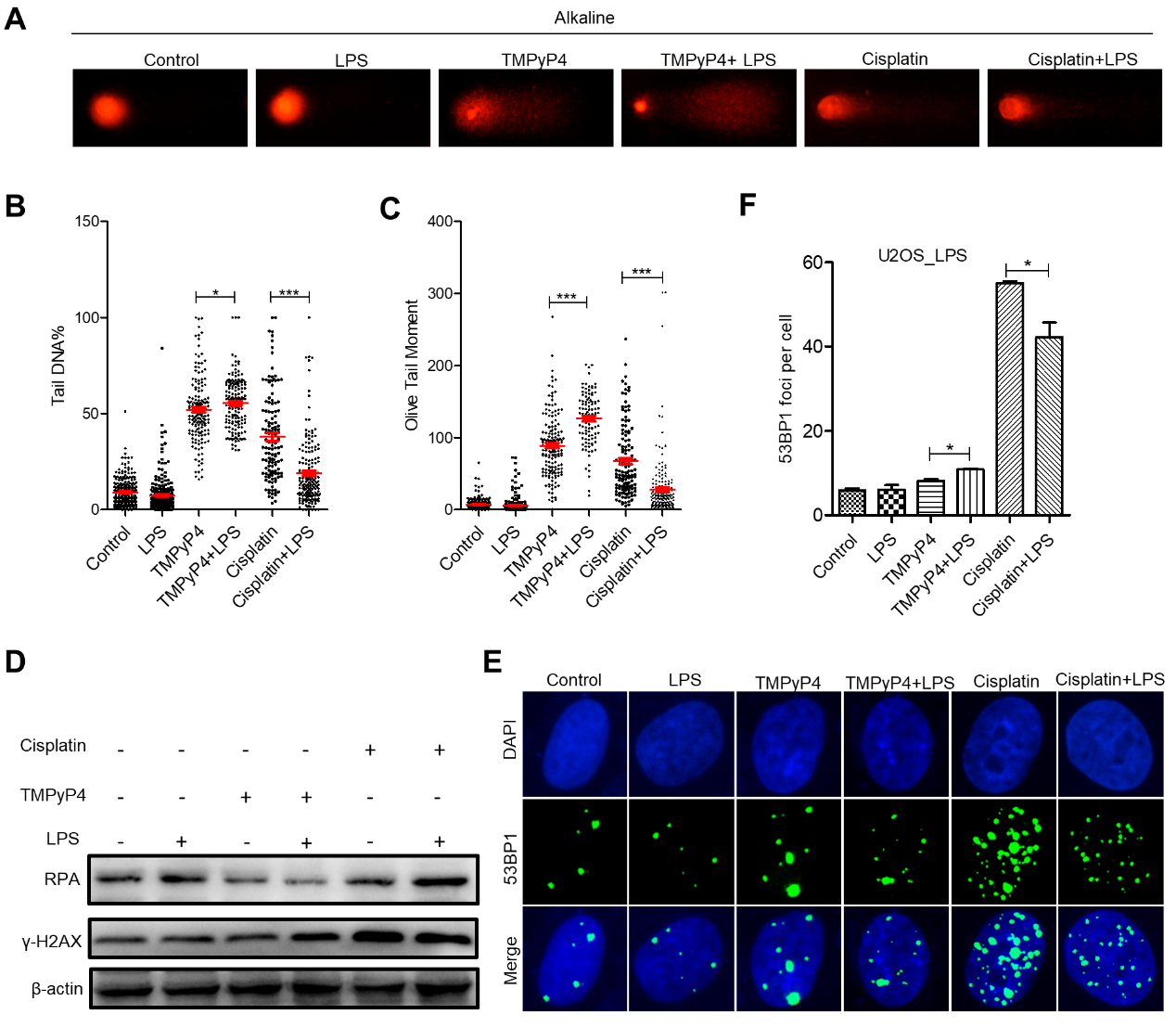


**Supporting Figure 2**. DNA damage and DNA damage response triggered by TMPyP4 combined with LPS. Cells were pretreated with indicated concentration of TMPyP4 or cisplatin for 24 hours and then were treated with or without LPS (1μg/ml) for 48 hours. **(A)**, The alkaline comet assay was performed to assess the DNA damage in U2OS cells. Magnification: 400×. **(B)**, **(C)** Quantification of A. For each group, ≥100 cells were examined. **(D)** Western Blot determination of abundance of γ-H2AX and RPA in U2OS cells. **(E)** IF visualization of 53BP1 foci in U2OS cells. Magnification: 400×. (F), Quantification of data in (E). For each group, ≥200 cells were examined. Values are average ± SD of three independent experiments. The statistical significance was calculated using the unpaired Student’s two-tailed *t*-test (**p* < 0.05, ***p* < 0.01, ****p* <0.001).


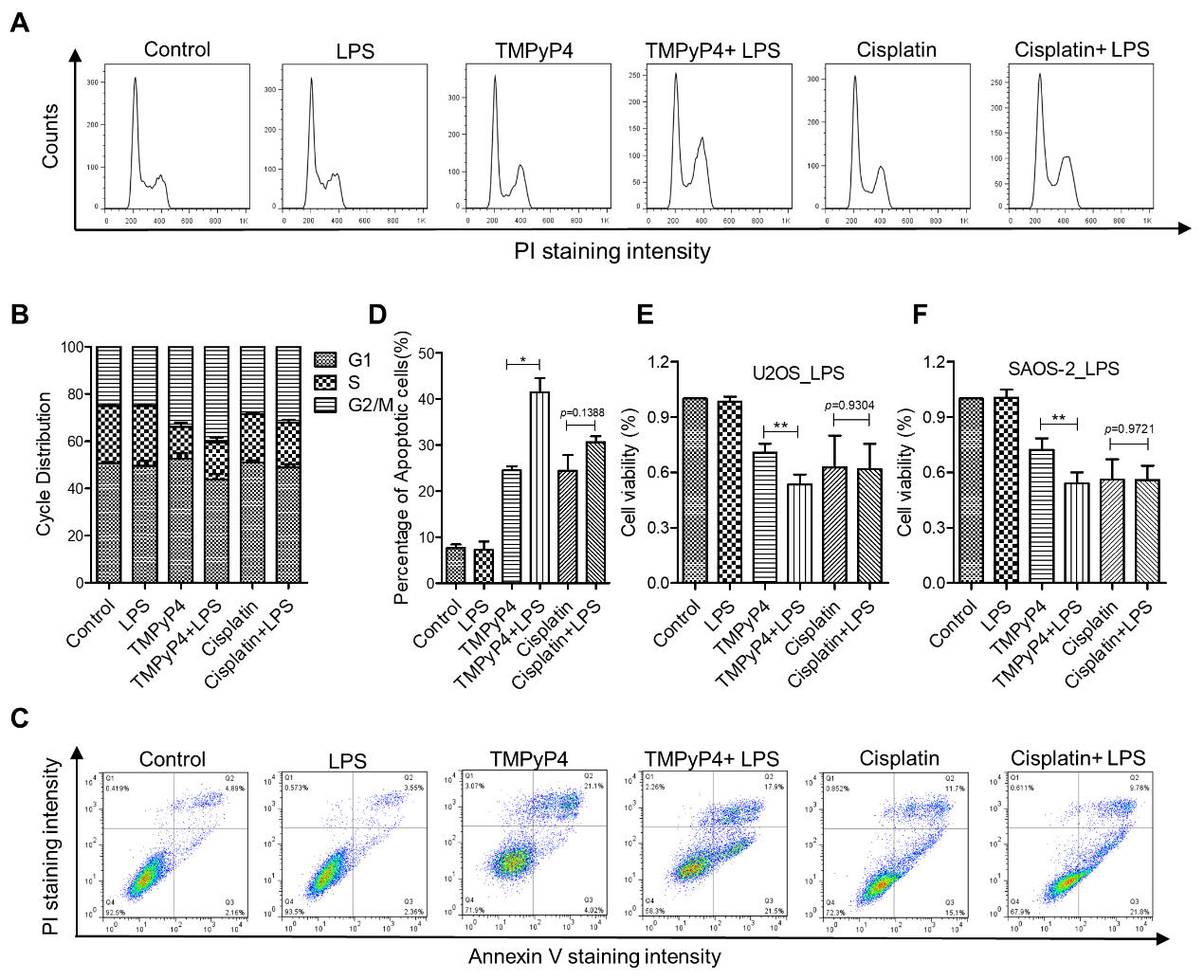


**Supporting Figure 3.** TMPyP4 inhibitors growth in U2OS and SAOS-2 cells in the inflammatory environment, and not change was observed in the group of cisplatin and LPS. Cells were challenged with indicated concentration of TMPyP4 or cisplatin in presence or absence of indicated LPS for indicated time. **(A)**, TMPyP4 combined with or without LPS blocked the U2OS cells in G2/M phase, **(B)**, Quantification of A. **(C)**, TMPyP4 combined with LPS induce severe apoptosis in U2OS cells. **(D)**, Quantification of C. **(E)** and **(F)**, TMPyP4 combined with LPS decrease OS cell viability by MTT assay. Data represent similar results from three independent experiments. The statistical significance was calculated using the unpaired Student’s two-tailed *t*-test (**p* < 0.05, ***p* < 0.01, ****p* <0.001).


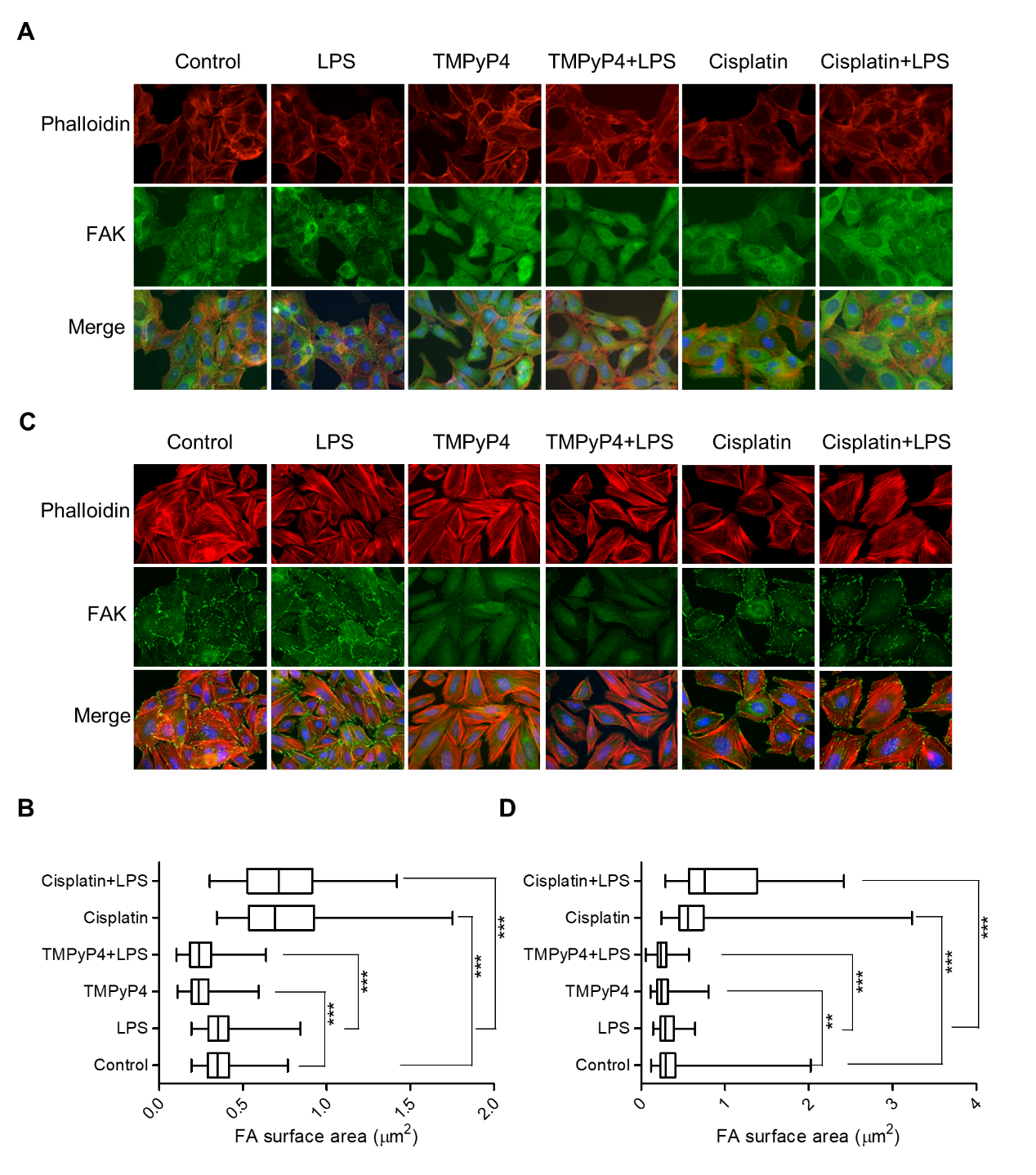


**Supporting Figure 4.** TMPyP4 combined with LPS decrease the expression and distribution of FAK in U2OS and SAOS-2 cells. Cells were challenged with indicated concentration of TMPyP4 or cisplatin in presence or absence of indicated LPS for indicated time. **(A)** and **(C)**, IF assay was performed to detect the distribution of FAK on U2OS or SAOS2 cells treated with indicated concentration of TMPyP4 or cisplatin with or without indicated LPS for indicated time. Magnification: 200×. **(B)** and **(D)**, Quantification of **(A)** and **(C)**. For each group, ≥200 cells were examined. The statistical significance was calculated using the unpaired Student’s two-tailed *t*-test (**p* < 0.05, ***p* < 0.01, ****p* <0.001).


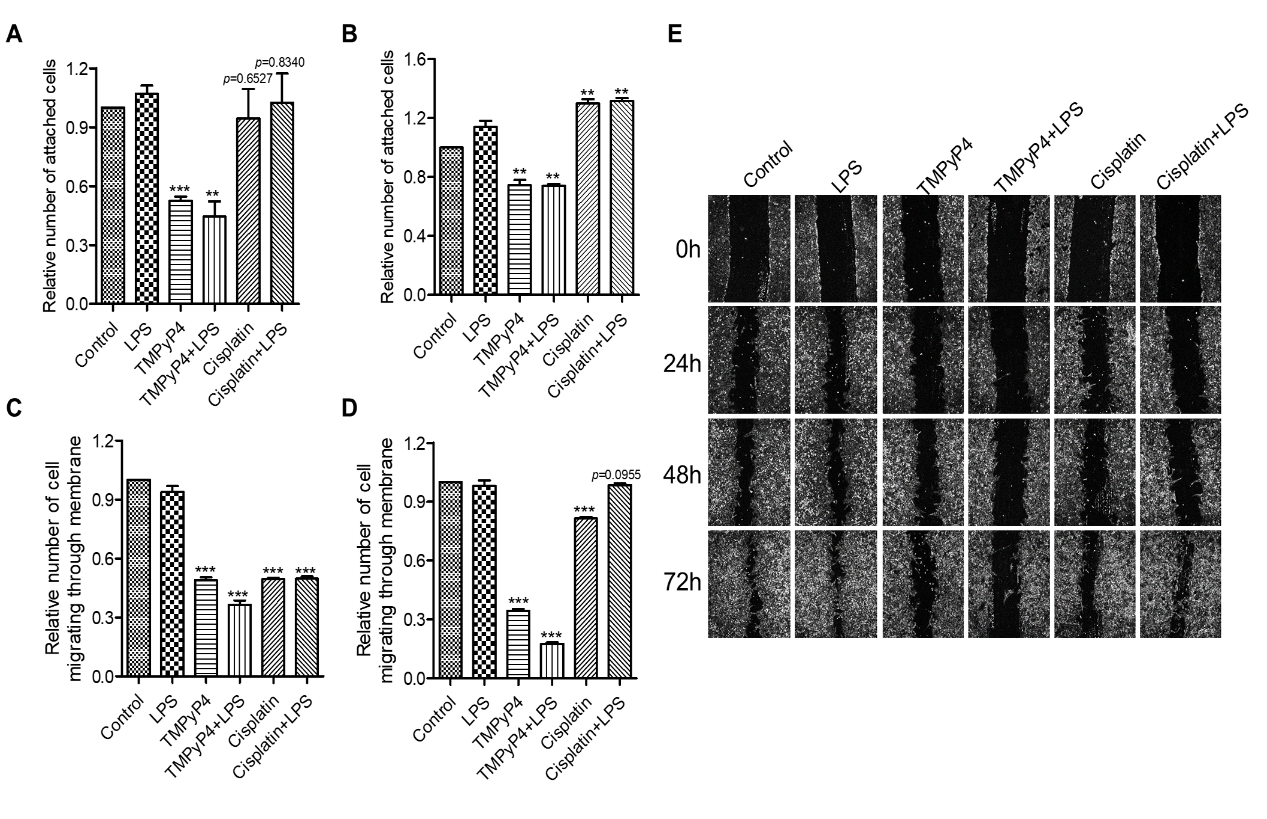


**Supporting Figure 5.** TMPyP4 alone or combined with LPS inhibitors the adhesion, invasion and migration abilities of OS cells, while the phenomenon was not observed in cisplatin or the group of cisplatin together with LPS. Cells were challenged with indicated concentration of TMPyP4 or cisplatin in presence or absence of indicated LPS for indicated time. **(A)** and **(B)**, TMPyP4 alone or combined with LPS decrease the adhesion abilities in U2OS and SAOS-2 cells, respectively. **(C)** and **(D)**, TMPyP4 or combined with LPS inhibitor the invasion abilities in U2OS and SAOS-2 cells, respectively. **(E)**, TMPyP4 alone or combined with LPS impeded cell migration in U2OS cells. Magnification: 40×.Values are average ± SD of three independent experiments. The statistical significance was calculated using the unpaired Student’s two-tailed *t*-test (**p* < 0.05, ***p* < 0.01, ****p* <0.001).
